# Healthy lifespan inequality: morbidity compression from a global perspective

**DOI:** 10.1007/s10654-023-00989-3

**Published:** 2023-04-07

**Authors:** Iñaki Permanyer, Francisco Villavicencio, Sergi Trias-Llimós

**Affiliations:** 1 Centre for Demographic Studies, Centres de Recerca de Catalunya (CERCA), Universitat Autònoma de Barcelona, 08193 Bellaterra, Spain; 2 ICREA, Passeig Lluís Companys 23, 08010 Barcelona, Spain; 3 Department of Economic, Financial and Actuarial Mathematics, University of Barcelona, Barcelona, Spain; 4 Interdisciplinary Centre on Population Dynamics, University of Southern Denmark, Odense, Denmark; 5 Department of International Health, Johns Hopkins Bloomberg School of Public Health, Baltimore, MD USA

**Keywords:** Ageing, Health inequalities, Longevity, Morbidity, Population health indicators

## Abstract

Current measures of population health lack indicators capturing the variability in age-at-morbidity onset, an important marker to assess the timing patterns of individuals’ health deterioration and evaluate the compression of morbidity. We provide global, regional, and national estimates of the variability in morbidity onset from 1990 to 2019 using indicators of healthy lifespan inequality (HLI). Using data from the Global Burden of Disease Study 2019, we reconstruct age-at-death distributions to calculate lifespan inequality (LI), and age-at-morbidity onset distributions to calculate HLI. We measure LI and HLI with the standard deviation. Between 1990 and 2019, global HLI decreased from 24.74 years to 21.92, and has been decreasing in all regions except in high-income countries, where it has remained stable. Countries with high HLI are more present in sub-Saharan Africa and south Asia, whereas low HLI values are predominant in high-income countries and central and eastern Europe. HLI tends to be higher for females than for males, and HLI tends to be higher than LI. Globally, between 1990 and 2019 HLI at age 65 increased from 6.83 years to 7.44 for females, and from 6.23 to 6.96 for males. Improvements in longevity are not necessarily accompanied by further reductions in HLI among longevity vanguard countries. Morbidity is compressing, except in high-income countries, where it stagnates. The variability in the ages at morbidity onset tends to be larger than the variability in lifespans, and such divergence broadens over time. As longevity increases worldwide, the locus of health inequality is moving from death-related inequalities to disease- and disability-centered ones.

**Supplementary Information:**

The online version contains supplementary material available at 10.1007/s10654-023-00989-3.

## Introduction

The ageing process that is unfolding across countries all over the world is an unprecedented landmark in human history: individuals worldwide can now expect to survive to ages that were deemed unattainable only a few decades ago [1–4]. However, despite the increases in life expectancy (LE) – the average number of years individuals are expected to live – attempts to reduce disease incidence and disability rates have been less successful [5]. In an ageing world, there are increasing concerns as to whether the extra years of life are spent in ‘good’ or in ‘less-than-good’ health [6], and there is a growing consensus that interventions to improve population health should not only focus attention on the quantity of years that individuals live, but also on their quality [7].

Surprisingly, current measures of population health have paid insufficient attention to the patterns of individuals’ health deterioration. While there are well-known indicators assessing how long are we expected to live in ‘good health’ *on average* – many health-adjusted life expectancy (HALE) indicators have been introduced in the literature for that purpose [6] – there is virtually no information about the *variability* in the ages at which individuals cease to be in good health. For example, a population in which all its members cease to be in good health at exactly age 65 is substantially different from a population in which 50% of its members cease to be in good health at age 55 and the other 50% at age 75 – a critical information for policy makers. The seminal work by Fries on the ‘compression of morbidity’ theory suggests that “morbidity is compressed into the shorter span between the increasing age-at-morbidity onset and the fixed occurrence of death” (p 133), and that “[t]he rectangularization of the survival curve may be followed by rectangularization of the morbidity curve and by compression of morbidity” (p 135) [8]. This theory naturally lends to the study of the variability in the ages-at-morbidity onset – a fundamental dimension of health that has been largely overlooked in the literature [9, 10].

We contribute to the ‘compression vs expansion of morbidity’ debate by using the recently proposed healthy lifespan inequality (HLI) indicators that measure the variability in healthy lifespans across individuals [10]. We estimate, for the first time, global, regional, and national levels and trends in HLI from 1990 to 2019, and compare the new indicator with currently existing population health measures such as LE, HALE and lifespan inequality (LI) [11]. There are several reasons why HLI can be considered a key indicator in health research that should be reported alongside traditional mortality and morbidity summary measures [10]. Improving population health goes beyond delaying death, and current societies are increasingly concerned not only with the rising prevalence of disease or disability, but also with rising health inequalities [12]. HLI indicators capture the heterogeneity in the underlying distribution of population health and can have important implications at the micro and macro levels. From the individuals’ perspective, HLI indicators can be used to study the variability in the ages-at-morbidity onset, a fundamental concept that measures the uncertainty in the timing of health deterioration [9, 10]. At the macro level, HLI indicators capture the heterogeneous characteristics of ageing populations, and provide crucial information for the design of social care and health provision programs.

## Methods

### Overview

**Fig. 1:**
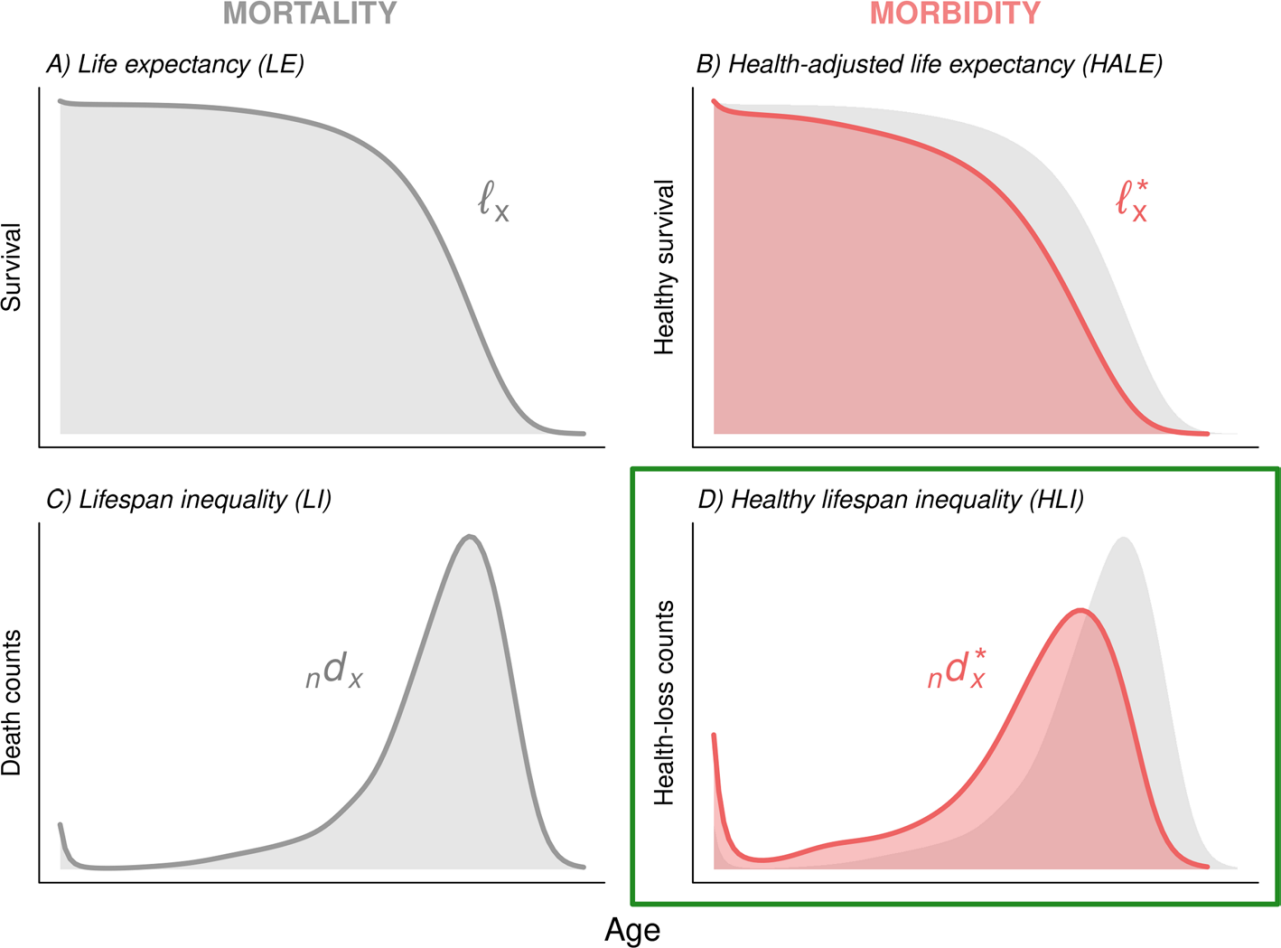
Diagram of key mortality-related (life expectancy and lifespan inequality) and morbidity-related (health-adjusted life expectancy and healthy lifespan inequality) indicators

We provide annual estimates on healthy lifespan inequality (HLI) for the overall population and above age 65 from 1990 to 2019 for 204 countries and territories using data from the Global Burden of Disease (GBD) Study 2019 [5, 13]. We also provide global and regional estimates using the same regional classification as GBD, which comprises seven super-regions and 19 regions (Supplementary Information [SI] Appendix 1). If no confusion arises, in the following we will refer to countries and territories as ‘countries’, and to super-regions and regions as ‘regions’. To obtain the different population health indicators, we first reconstructed ‘mortality curves’ (commonly referred to as ‘survival curves’) based on life table data (Fig. 1 panel A). Next, we reconstructed ‘morbidity curves’ using age-specific remaining HALE estimates from GBD (Fig. 1 panel B). Morbidity curves measure survival probabilities in good health. Finally, from the mortality and morbidity curves we obtained the corresponding age-at-death (Fig. 1 panel C) and age-at-morbidity onset (Fig. 1 panel D) distributions, from which we calculated LI and the new HLI indicators [10].

### Data

The Global Burden of Disease Study 2019 reports global, regional, and national estimates on age- and sex-specific probabilities of dying and age- and sex-specific remaining LE in 5-year age groups from 1950 to 2019 [14]. These data can be used to reconstruct mortality curves for all region- and country-years using standard life table techniques. Recently, GBD has also been publishing estimates of age- and sex-specific HALE for all countries from 1990 to 2019 [5, 13]. These estimates are obtained using models that incorporate estimates of years lived with disability, life tables, and standard demographic methods [5] – but the underlying morbidity curves remain unknown.

### Reconstructing mortality and morbidity curves

Using data on age-specific probabilities of dying and age-specific remaining LE, for each country/region, year, and sex we calculated the age-specific average person-years lived in each age interval by individuals dying in that interval (the ${}_{n}{a}_{x}$ values) and the remaining columns of the life table, including the mortality/survival curves $\ell _{x}$ (Fig. 1 panel A). Next, we used the ${}_{n}{a}_{x}$ values from each setting, in combination with the age-specific HALE estimates, to reconstruct the corresponding morbidity curves $\ell _{x}^{*}$ (Fig. 1 panel B). Additional details on the reconstruction of mortality and morbidity curves are found in the SI (Appendix 2).

### Calculating inequality

For each country/region, year, and sex we took first differences from the mortality and morbidity curves to derive the corresponding age-at-death ($_{n} d_{x} = \ell _{x} - \ell _{{x + n}}$, Fig. 1 panel C) and age-at-morbidity onset ($_{n} d_{x}^{*} = \ell _{x}^{*} - \ell _{{x + n}}^{*}$, Fig. 1 panel D) distributions (SI Appendix 2). From these distributions, we measured the corresponding levels of LI and HLI using the standard deviation (SI Appendix 3.1), an indicator measuring the spread of a distribution that has been widely used as a measure of lifespan inequality [15, 16]. Our findings proved robust when compared to those obtained using other popular inequality measures, such as the coefficient of variation or the Gini index given the strong correlation among them (SI Appendix 3.2). When the indicators proposed in the paper are computed for ages above 65, we add the number 65 to the acronyms used to refer to them (i.e., LE65, HALE65, LI65 and HLI65).

### Measuring morbidity compression

To assess whether morbidity is ‘expanding’ [17] or ‘compressing’ [8], we adopted the approach proposed by Fries [8], who suggested evaluating the extent to which morbidity curves become increasingly rectangular over time. To do so, we followed Wilmoth and Horiuchi [18] and measured whether the age-at-morbidity onset distributions (${}_{n}{d}_{x}^{*}$) become more or less concentrated (i.e., whether HLI increases or decreases over time).

### Uncertainty analysis

We assessed the uncertainty of the LI and HLI estimates based on the uncertainty of the input data from GBD. Uncertainty was obtained by sampling from the corresponding uncertainty intervals (UIs) of LE, death probabilities, and HALE reported by GBD on Monte Carlo simulations (SI Appendix 3.3), adapting an analogous approach used elsewhere [19]. We report 80% UIs rather than 95% UIs because of the substantial uncertainty inherent in HALE estimates [13, 19]. Intervals based on higher uncertainty levels would not constitute useful and meaningful summary measures.

We followed the GATHER guidelines for global health estimates and included the GATHER checklist for transparency and replicability (SI Appendix 4). We carried out all our analyses using the open-source statistical software R (version 4.1.1) [20]. To reduce computing time we implemented parallel processing using the R package ‘doParallel’ (version 1.0.17) [21].

## Results

### Levels and trends in healthy lifespan inequality (HLI)

**Fig. 2:**
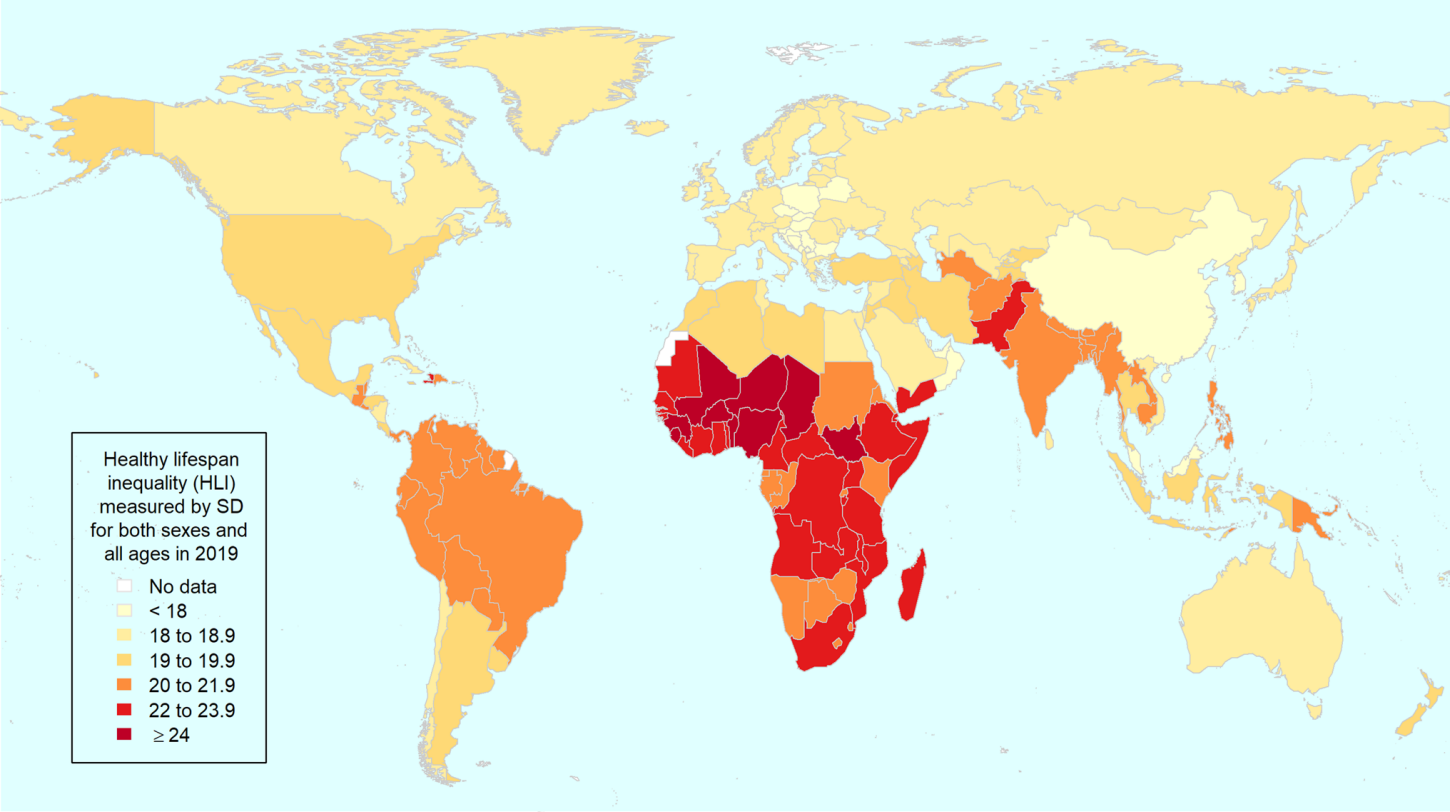
World map with national healthy lifespan inequality levels in 2019, both sexes combined. Source: Authors’ elaboration based on data from GBD [5, 13]

In 2019, the regions with the lowest HLI values are southeast Asia, east Asia and Oceania, central Europe, eastern Europe and central Asia, and high-income countries; at the opposite end, the highest HLI is observed in sub-Saharan Africa and south Asia (Table 1). The countries with the highest HLI values (above 22 years) are mostly concentrated in sub-Saharan Africa (for instance Mali, Niger, Nigeria, Burkina Faso, and Chad), but high HLI values are also observed in Haiti, Pakistan, and Yemen (Fig. 2). The mid-range of the HLI distribution (19−22 years) is mainly represented by countries from Latin America and Caribbean like Brazil, from south Asia (India and Bangladesh), from sub-Saharan Africa (Rwanda, Kenya, and Congo), and from north Africa and Middle East (Sudan and Afghanistan). At the bottom of the distribution, the lowest HLI values (below 19) are mostly found in high-income countries, but also in China and central and eastern Europe (Fig. 2).

**Table 1:** Global and regional estimates of healthy lifespan inequality by sex in 2019. Healthy lifespan inequality measured as the standard deviation of the corresponding age-at-morbidity onset distribution (values between parenthesis denote 80% uncertainty intervals)

	Females	Males	Both sexes
*Global*	22.10 (17.61−26.54)	21.65 (17.83−25.36)	21.92 (17.79−25.80)
**Central Europe, eastern Europe and central Asia**	18.83 (14.64−22.01)	18.57 (15.28−21.35)	18.97 (15.37−22.17)
Central Asia	19.20 (15.17−22.91)	18.87 (15.48−22.26)	19.17 (15.46−22.69)
Central Europe	17.70 (13.59−20.65)	17.16 (13.51−19.82)	17.60 (13.88−20.40)
Eastern Europe	18.43 (14.43−21.32)	18.25 (15.09−20.87)	18.69 (15.24−21.31)
**High-income countries**	19.35 (14.96−22.46)	18.46 (14.63−21.52)	18.95 (14.86−21.91)
Australasia	19.15 (14.64−22.22)	18.54 (14.40−21.75)	18.87 (14.55−21.98)
High-income Asia Pacific	18.55 (14.03−21.65)	17.39 (13.68−20.56)	18.05 (14.03−21.23)
High-income North America	19.59 (15.27−22.59)	18.99 (15.18−21.91)	19.33 (15.22−22.34)
Southern Latin America	19.21 (15.01−22.41)	18.70 (15.01−21.96)	19.04 (15.06−22.44)
Western Europe	18.89 (14.47−22.03)	17.72 (13.92−20.73)	18.35 (14.27−21.45)
**Latin America and Caribbean**	20.62 (16.13−24.36)	20.87 (17.07−24.45)	20.82 (16.68−24.45)
Andean Latin America	20.50 (15.76−24.32)	20.64 (16.08−24.70)	20.59 (15.89−24.37)
Caribbean	22.67 (18.26−26.77)	22.50 (18.35−26.26)	22.62 (18.47−26.63)
Central Latin America	20.01 (15.57−23.26)	20.51 (16.55−24.00)	20.31 (16.18−23.88)
Tropical Latin America	20.94 (16.52−24.95)	21.16 (17.47−24.50)	21.16 (17.14−25.01)
**North Africa and Middle East**	20.32 (15.90−24.11)	20.24 (16.18−24.08)	20.28 (16.09−24.10)
**South Asia**	21.78 (17.30−25.85)	21.24 (17.08−25.09)	21.50 (17.37−25.24)
**Southeast Asia, east Asia and Oceania**	18.64 (14.10−22.16)	18.57 (14.60−22.15)	18.66 (14.53−22.10)
East Asia	17.49 (12.97−20.63)	17.33 (13.29−20.70)	17.47 (13.41−20.59)
Oceania	21.58 (17.19−25.53)	21.42 (17.40−25.16)	21.49 (17.29−25.43)
Southeast Asia	19.69 (15.38−23.39)	19.73 (15.95−23.34)	19.79 (15.78−23.38)
**Sub-Saharan Africa**	23.96 (20.39−27.47)	23.76 (20.77−26.66)	23.89 (20.71−27.12)
Central sub-Saharan Africa	22.85 (18.83−26.67)	22.44 (18.68−25.76)	22.70 (18.91−26.54)
Eastern sub-Saharan Africa	22.97 (19.26−26.56)	22.70 (19.54−25.85)	22.86 (19.41−26.11)
Southern sub-Saharan Africa	22.52 (19.13−25.75)	21.74 (19.02−24.43)	22.24 (19.19−25.26)
Western sub-Saharan Africa	25.10 (21.57−28.64)	25.20 (22.05−28.18)	25.16 (21.89−28.34)

**Fig. 3:**
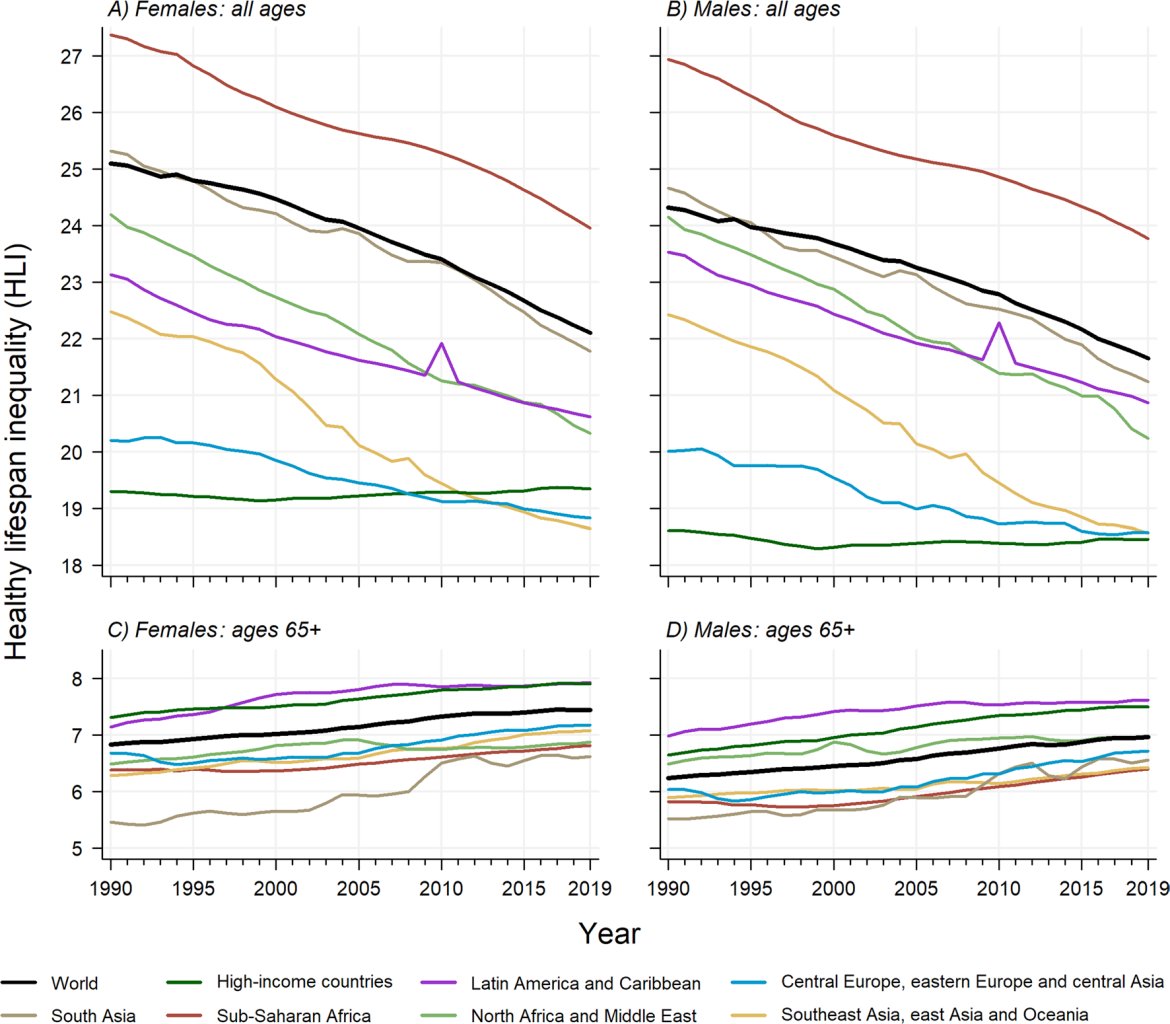
Global and regional time trends in healthy lifespan inequality by sex, for all ages (panels A and B) and for ages above 65 (panels C and D) from 1990 to 2019. Source: Authors’ elaboration based on data from GBD [5, 13]

Between 1990 and 2019, global HLI levels declined from 25.09 years (80% uncertainty interval [UI] 21.50−28.46) to 22.10 (17.61−26.54) for females, from 24.32 (21.51−27.09) to 21.65 (17.83−25.36) for males, and from 24.74 years (21.59−27.74) to 21.92 (17.79−25.80) for both sexes combined. Steady declines have also been observed across all world regions except for high-income countries, which have remained stable between 1990 and 2019 with HLI levels hovering around 19.3 years for females and 18.4 for males. In most regions, HLI levels are larger for females than for males (e.g., sub-Saharan Africa, south Asia, and central Europe, eastern Europe and central Asia), except for Latin America and the Caribbean (Fig. 3 panels A and B).

For ages above 65, we find increasing HLI65 trends across the board (Fig. 3 panels C and D). Globally, HLI65 goes from 6.83 years (80% UI 6.40−7.28) in 1990 to 7.44 (6.91−8.03) in 2019 for females, and from 6.23 (5.93−6.58) to 6.96 (6.56−7.41) for males. Both for females and males, the highest HLI65 levels are observed for Latin America and Caribbean and high-income countries. At the other end, south Asia and sub-Saharan Africa stand out as the regions with lowest HLI65 levels. Once again, HLI65 levels tend to be higher for females than for males. All the global, regional, and national LI and HLI estimates, and the corresponding 80% uncertainty intervals, are available on GitHub (see SI Appendix 5 for details).

### HLI and other population health measures

**Fig. 4:**
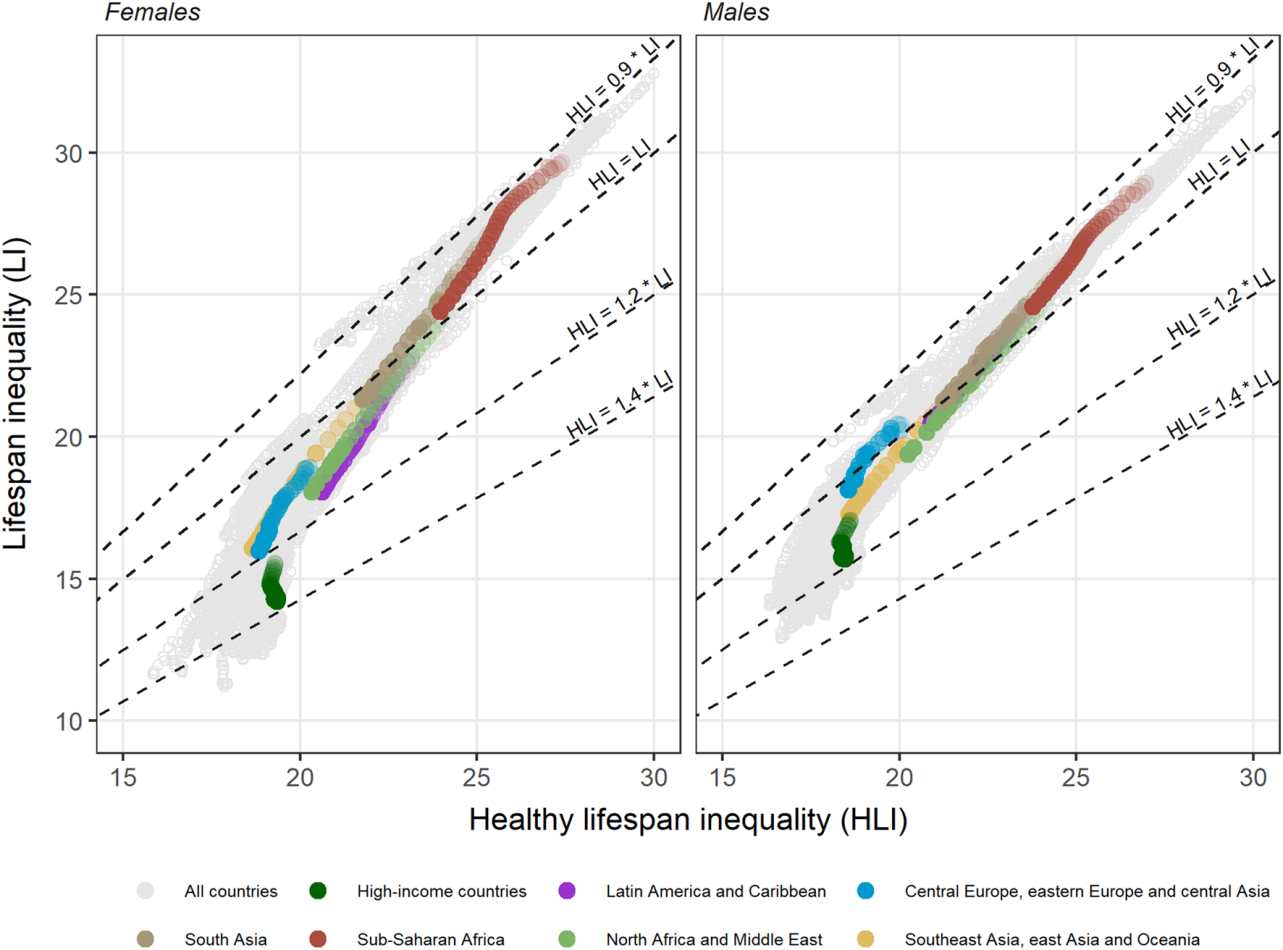
Relationship between healthy lifespan inequality and lifespan inequality by sex between 1990 and 2019. For each region, the lightest color corresponds to 1990 and darkness increases over years up to 2019. Source: Authors’ elaboration based on data from GBD [5, 13, 14]

A visual inspection of the association between LI and HLI reveals that these two measures are positively correlated, and their values follow clear temporal and geographical patterns (Fig. 4). Both HLI and LI tend to decline over time, even though the latter declines faster. As we approach 2019, for most country-year observations the values of HLI are larger than those of LI, being sub-Saharan African countries the only exception. This is especially noticeable when analyzing global and regional trends in the ratio between HLI and LI (SI Appendix 6): increasing trends in all regions are observed but point estimates in sub-Saharan Africa remain below one for the entire 1990–2019 period. In high-income countries (located at the lower end of the cloud of data points in Fig. 4), the HLI/LI ratio reaches 1.36 (80% UI 1.05–1.58) for females, meaning that HLI levels are 36% higher than those of LI (SI Appendix 6). Worldwide, in 2019 HLI is higher for females than for males in 153 (75.0%) of the 204 countries and territories analyzed, in contrast with LI, that is higher for males in 183 (89.7%) of the cases. HLI is higher than LI in 321 (78.7%) of the 408 country-sex combinations.

**Fig. 5:**
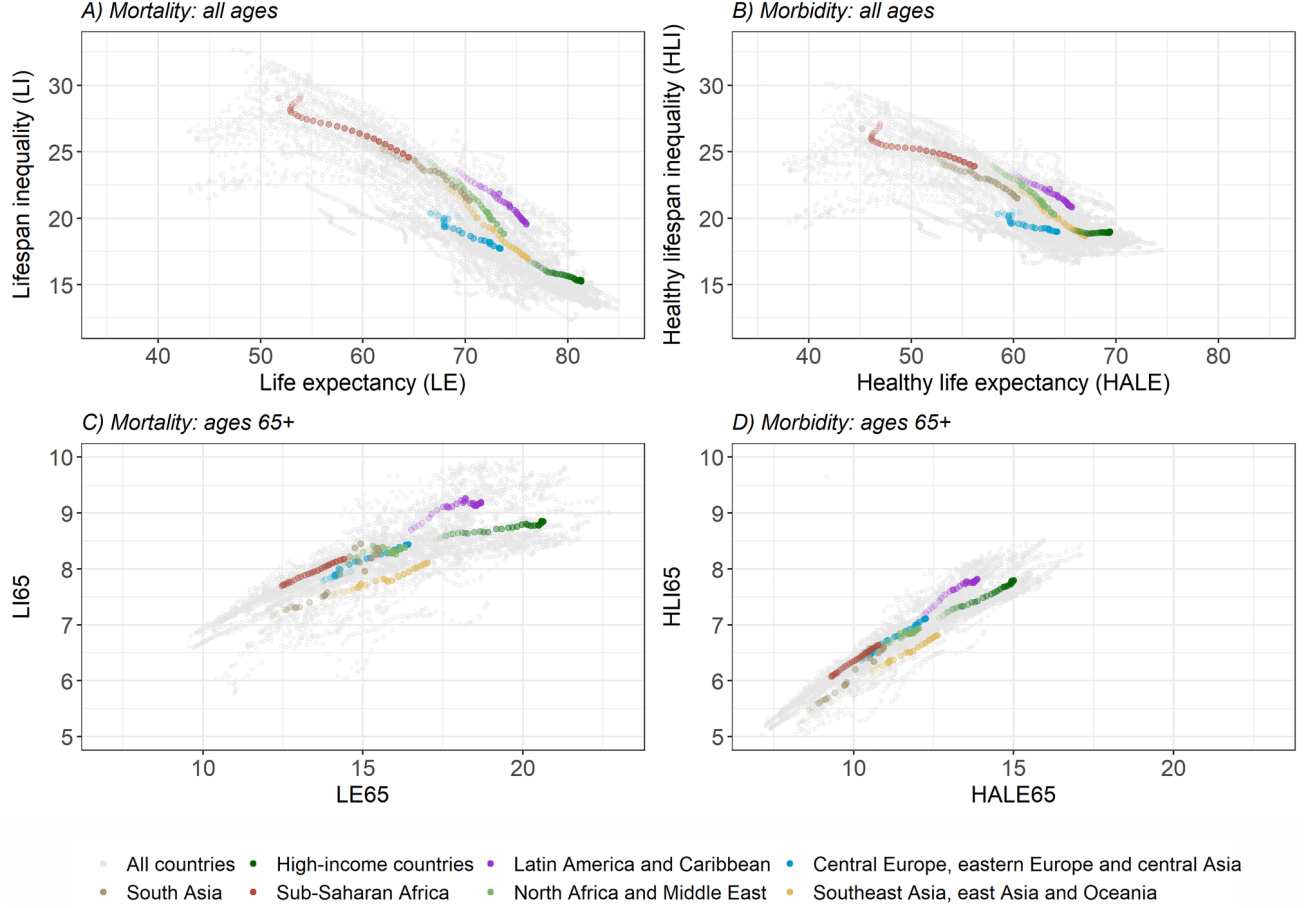
Relationship between life expectancy - lifespan inequality and health-adjusted life expectancy - healthy lifespan inequality for females between 1990 and 2019, considering all ages (panels A and B) and ages above 65 (panels C and D). Source: Authors’ elaboration based on data from GBD [5, 13, 14]

The overall association between average health (as measured by LE and HALE) and health inequality (as measured by LI and HLI) is negative, showing that higher longevity and healthy longevity tend to be associated with lower variability in lifespans and healthy lifespans within countries, respectively (Fig. 5 panels A and B). While the relationship between LE and LI is strong and negative (Fig. 5 panel A, the slope of the linear trend is − 0.43 with ${R}^{2}=$ 0.747), it is weaker and flatter between HALE and HLI (Fig. 5 panel B, slope of − 0.28 and ${R}^{2}=$ 0.590). The negative relationship between the later tends to disappear (or even reverse to positive) when the values of HLI hover around 18, as it is the case in high-income countries and central Europe, eastern Europe and central Asia. That is, despite continued improvements in HALE, HLI levels seem to have reached a lower bound where no further reductions (or indeed increases) are observed – a pattern that does not occur when comparing LE and LI.

For ages above 65 years, the association between average health and health inequality reverses to positive across all world regions (Fig. 5 panels C and D). That is, when conditioning upon survival to 65, higher average longevity is associated with more variability in ages-at-death, and higher healthy longevity is associated with greater variability in the ages at which morbidity starts. While there is a great deal of cross-country variability in the relationship between LE65 and LI65 (Fig. 5 panel C), the association between HALE65 and HLI65 is much tighter (Fig. 5 panel D). In addition, LI65 levels tend to be larger than those of HLI65, but the latter increase at a faster pace.

## Discussion

### Summary

Healthy lifespan inequality (HLI) indicators measure the variability in age-at-morbidity onset, so they can be used to assess the time patterns of individuals’ health deterioration.

This study documents, for the first time, the levels and trends in HLI indicators across regions and countries all over the world for a period of 30 years, from 1990 to 2019. Global HLI declined from 24.74 years (80% UI 21.59−27.74) in 1990 to 21.92 (17.79−25.80) in 2019. Generalized declines are observed across regions, except for high-income countries, where HLI has remained stagnant – and even increasing slightly since 2000. At ages above 65 years, HLI65 increased over the study period in all seven super-regions. Importantly, the values of HLI tend to be substantially larger than those of LI in all regions except for sub-Saharan Africa, and such differences increase over time. Higher values of LE tend to be associated with lower values of LI, but the relationship between HALE and HLI is considerably weaker (especially among longevity vanguard countries). In general, males tend to exhibit higher levels of LI than females, but the opposite is observed with HLI.

### Interpretation

Our results suggest that, while the variability in the ages at which morbidity starts has decreased in most high-mortality countries, it has remained constant or has even increased for an ever-growing number of countries, especially among the longevity vanguards. In those countries, even if age at death is retreating and becoming increasingly concentrated at older ages [11, 15, 22–24], it is less clear whether morbidity onset is also being shifted towards older ages [5, 25, 26]. In general, improvements in reducing mortality rates for most causes have not been matched by similar declines in disability rates, which have either been stagnant or have increased for several causes, like diabetes or mental and substance use disorders [7]. Our findings cohere with previous studies exploring mortality and morbidity dynamics from a global perspective – while expanding and complementing those analyses in several directions.

In this paper, we find that the variability in the ages at which morbidity starts (HLI) can be much larger than the variability in the ages at death (LI), and such difference broadens over time. When this happens, the range of ages in which most individuals’ health deteriorates becomes wider than the range of ages in which most individuals die – a key finding with fundamental implications for planners aiming at improving population health and reducing health inequalities. For instance: retirement or health care policies exclusively based on (increasing) trends in life expectancy might miss the mark and have deleterious social consequences if the corresponding morbidity onset distribution widens over time. Indeed, HLI levels are estimated to be up to 36% higher than LI among women in high-income countries. The only exception to this general pattern is found in sub-Saharan Africa, where LI levels are slightly higher than those of HLI – even though they become increasingly similar over time (see Fig. 4 and SI Appendix 6). This might be attributable to the high levels of child mortality in the region [27] (which generate bimodal age-at-death distributions with high levels of lifespan inequality) together with the fact that the incidence of morbidity is relatively low at those younger ages. Further research is needed to examine what are the specific age groups and causes of death, diseases or conditions driving the trends we document.

The strong and negative association between country-specific LE and LI estimates (Fig. 5 panel A) has been widely documented [22–24, 28]. It suggests that the normatively desirable goals of increasing countries’ average longevity and reducing age-at-death inequality can be achieved simultaneously. However, a different picture emerges when inspecting the relationship between HALE and HLI (Fig. 5 panel B). There are many countries and regions experiencing increases in HALE concomitantly with declines in HLI – thus suggesting that a ‘compression’ [8] of morbidity is occurring in those places – (e.g., sub-Saharan Africa, south Asia, north Africa and Middle East, or other regions at the bottom of the corresponding distributions with plenty of room for improvement). At the same time, there seems to be a threshold above which further gains in HALE are not necessarily accompanied with HLI reductions. This is the case for a considerable number of countries – particularly those from central Europe, eastern Europe and central Asia and, specially, high-income countries – where it is not clear whether morbidity is ‘expanding’ [17] or ‘compressing’ [8]. Thus, while high-mortality countries have been generally successful in increasing (healthy) longevity and simultaneously reducing (healthy) lifespan inequality, low-mortality countries have made no further progress in reducing the variability in healthy lifespans. Such stagnation might be the outcome of forces pushing in opposite directions. On the one hand, improvements in living standards or the promotion of healthier lifestyles (e.g., better diets, regular exercise, avoiding alcohol consumption or smoking) [29, 30] can postpone the deterioration of individuals’ health. On the other hand, the implementation of prevention and screening programs that decrease the age at diagnosis of important diseases (e.g., cancers or mental disorders) [31, 32] as well as persistent and increasing socioeconomic inequalities [33] can contribute to widen age-at-morbidity onset distributions.

These troubling trade-offs between health equality and efficiency become even more pronounced when inspecting trends at ages above 65 years. Our findings indicate that countries’ overall success in increasing LE65 and HALE65 inevitably comes at a cost: a simultaneous increase in both LI65 and HLI65, indicating greater heterogeneity among the elder population. These results cohere with recent studies reporting increasing trends in lifespan inequality at age 75 and a positive relationship between LE65 and LI65 [23], and suggest that the variability in morbidity onset at older ages (HLI65) is going in the same direction. Indeed, Seaman et al. also find that the variability in the age at morbidity onset in Denmark (as measured by the age at first hospital admission among adults aged 60 and above) also tends to increase from the 1990s onwards [9]. Unfortunately, the approach followed in that study cannot be replicated at a global scale because of its reliance on hospital admission data – which is difficult to access and is not easily comparable across countries. Likewise, our findings on increasing health variability among the elder are in line with recent studies documenting an increase in the diversification of the causes from which individuals die in low-mortality countries – especially among individuals dying at ages above 50 [34]. Put together, these studies posit that, as mortality is pushed towards increasingly higher ages, the health profiles of the survivors are increasingly diverse (i.e., with an increasingly heterogeneous mix of robust and frail individuals).

The findings presented here consistently point towards a compositional shift in health inequalities within countries: as longevity increases, morbidity-related inequalities are gaining prominence with respect to mortality-related ones. That is, generalized improvements in living standards, medical innovations, the spread of technological breakthroughs and public health policies might have contributed to reduce mortality-related inequalities across world countries. This is reflected in the decreasing inequalities in basic survival indicators like life expectancy at birth, mostly driven by the reduction of infant and child mortality [5, 27]. However, these improvements have in turn contributed to the emergence of new layers of morbidity-related inequalities among adults at older ages, often to the advantage of privileged countries or socio-economic groups [10, 35]. Stated otherwise: the same structural improvements that have contributed to increase the survival chances of the worse-off have in turn delayed the emergence of health-related inequalities to older ages. This is yet another instance of the successive waves of convergence-divergence cycles in health stipulated by the health transition theory suggested by Frenk et al. [36] and later adopted by Vallin and Meslé [37].

Furthermore, our results shed new light on the male-female health and mortality differences. Women tend to live longer, and the length of their lives are less unequally distributed than men (Fig. 4) [6]. However, the female advantage is less obvious, or even disappears, when looking at the length of healthy life. It is well known that the sex gaps in HALE are considerably smaller than those in LE [5, 6]. As regards healthy lifespan inequality, we find that in approximately three quarters of our country-year observations female HLI levels are higher than those of males. These findings suggest that, not only women spend longer fractions of their lives in less-than-good health as postulated in the ‘health-survival paradox’ [6, 38], but that they tend to face greater uncertainty than men regarding the ages at morbidity onset. Further research on the determinants of the increasing sex-differences in HLI is also needed.

### Limitations

Our paper has several limitations. First, our estimates are exclusively based on period life tables. Unfortunately, longitudinal methods can only be applied in a highly reduced number of data-rich countries. Hence, we rely upon the synthetic cohort approach in which individuals are subject to period-specific mortality and morbidity conditions along their lifetimes, something that is customarily used in the estimation of LE, LI and HALE indicators. Second, the methods used to generate age-at-morbidity onset distributions implicitly rely on the assumption that individuals cannot recover from their ‘less-then-healthy’ status. While somewhat unrealistic, this is the simplifying assumption underlying the Sullivan method [39], which under mild regularity conditions is generally acceptable for monitoring long-term trends in HALE [40], and has been widely used for estimating HALE indicators [6], also by GBD [5]. In addition, the definition of HALE is exclusively based on the prevalence and severity of diseases and health conditions, but fails to take into consideration other, more holistic, dimensions of health (like self-reported health, or pain and discomfort levels) typically included in other approaches, like the EQ-5D measure of the EuroQol Group [41]. Third, the quality of the mortality and, specially, morbidity data varies considerably across countries (an issue that is partially attributable to the different sampling strategies followed to obtain health information), which is reflected in the uncertainty intervals of our HLI estimates. All our analyses are based on levels and trends of the point estimates, but conclusions should be formulated with caution given their uncertainty [19]. Lastly, the outbreak of Covid-19 pandemic has not been included in our analyses. A comprehensive study on the potential impact that the pandemic might have on the population health indicators investigated here is extremely important, but should probably await better data.

## Conclusion

Previous studies investigating the ‘compression vs expansion of morbidity’ debate have almost exclusively relied on the comparison of indicators measuring *average* longevity (i.e., LE) against indicators measuring the *average* number of years spent in good health (HALE) [6, 25]. This ‘average-based approach’ has many limitations, as it disregards the patterns of health deterioration among individuals. As emphasized by Fries [8] more than four decades ago, the analysis of variation (not of average values) is fundamental to investigate whether, and to what extent, the emergence of diseases is postponed to increasingly older ages. Notwithstanding the limitations of our approach, this paper sheds new light into a longstanding debate with fundamental implications for our understanding of contemporary health dynamics around the globe.

Most attempts at explaining the drivers of population ageing are framed within the epidemiological transition theory [42] or some of its variations – like the fourth stage added to Omran’s initial theory [43–45] (the so-called “Age of delayed degenerative diseases”). Among the major critiques directed against it, scholars have highlighted its overemphasis on mortality (e.g., causes of death), thus giving insufficient attention to morbidity and quality of life [46]. The findings reported in this paper not only lend support to the idea that prospective theories on population ageing should give a more prominent role to morbidity, but also highlight the importance of moving beyond averages to study the age and sex patterns in which morbidity onset affect individuals across populations. Whether further delays in the ages at which individuals die pose a threat to the sustainability of the health, pension, and welfare systems not only depends on the extent to which these extra years of life are lived free of disease, disability and/or physical and mental impairment, but also on the timing patterns of individuals’ health deterioration.

The results reported in this study uncover a surprisingly overlooked layer of inequality that cannot be observed with traditional population health measures such as LE, HALE or LI, and have substantive implications. Inter alia, they reveal that mortality inequalities within countries are becoming less prominent than its morbidity counterparts. As longevity increases worldwide, the locus of health inequality over the lifespan is gradually moving from death-related inequalities to disease- and disability-centered ones – a compositional shift in health inequality that should be taken into consideration in the elaboration of prospective public health policies.

## Electronic supplementary material

Below is the link to the electronic supplementary material.

Supplementary file1 (PDF 875 kb)

## Data Availability

The source code to replicate the analyses, input data, and results are publicly available for research purposes on the GitHub repository https://github.com/panchoVG/HLI).
