# Supplementary material for: Healthy lifespan inequality: morbidity compression from a global perspective
Source: Eur J Epidemiol. 2023 Apr 7;38(5):511–21. doi: 10.1007/s10654-023-00989-3 (PMC10080172; doi:10.1007/s10654-023-00989-3)
Supplement: Supplementary file 1 — Supplementary file1 (PDF 875 kb) [file 10654_2023_989_MOESM1_ESM.pdf]

Supplementary information for

Healthy lifespan inequality: morbidity compression from a global perspective

Iñaki Permanyer

Francisco Villavicencio

Sergi Trias-Llimós

March 2023

Table of contents

Appendix 1. Regional and country classification ... 2

Table S1. Regional classification ... 2

Table S2. Country classification ... 3

Appendix 2. Reconstruction of mortality and morbidity curves ... 7

2.1 Mortality curves ... 7

2.2 Morbidity curves ... 7

2.3 Adjustments of the nax values ... 9

Appendix 3. Inequality measures and uncertainty ... 11

3.1 The standard deviation as a measure of inequality ... 11

3.2 Other inequality measures ... 11

3.3 Uncertainty estimation ... 13

3.4 Transparency and replicability ... 14

Appendix 4. The GATHER checklist ... 15

Appendix 5. Lifespan inequality and healthy lifespan inequality estimates ... 16

Appendix 6. Global and regional HLI/LI ratios ... 17

References ... 18

Appendix 1. Regional and country classification

We used the same regional and country classification as the Global Burden of Disease (GBD) Study 2019.[1] This comprises 204 countries and territories, 19 regions, and seven super-regions. Estimates at the global/world level are also provided.

The classification can be visualized at https://www.iapb.org/learn/vision-atlas/about/definitions-and-regions/ (accessed on May 2, 2022) and is summarized in the tables below. These tables have the following variables/columns:

- ISO3: Three-letter country codes (only for countries/territories)
- location_id: Location number assigned by GBD
- location_name: Name of the super-region, region, and country/territory
- Type: Whether the location is a super-region or a region
- Super-region: Super-region at which a country/territory belongs
- Region: Region at which a country/territory belongs

Table S1. Regional classification

location_id	location_name	Type
1	**Global/World**	
4	**Southeast Asia, east Asia and Oceania**	Super-region
5	East Asia	Region
9	Southeast Asia	Region
21	Oceania	Region
31	**Central Europe, eastern Europe and central Asia**	Super-region
32	Central Asia	Region
42	Central Europe	Region
56	Eastern Europe	Region
64	**High-income countries**	Super-region
65	High-income Asia Pacific	Region
70	Australasia	Region
73	Western Europe	Region
96	Southern Latin America	Region
100	High-income North America	Region
103	**Latin America and Caribbean**	Super-region
104	Caribbean	Region
120	Andean Latin America	Region
124	Central Latin America	Region
134	Tropical Latin America	Region
137	**North Africa and Middle East**	Super-region/Region
158	**South Asia**	Super-region/Region
166	**Sub-Saharan Africa**	Super-region
167	Central sub-Saharan Africa	Region
174	Eastern sub-Saharan Africa	Region
192	Southern sub-Saharan Africa	Region
199	Western sub-Saharan Africa	Region

Table S2. Country classification

ISO3	location_id	location_name	Super-region	Region
AFG	160	Afghanistan	North Africa and Middle East	North Africa and Middle East
AGO	168	Angola	Sub-Saharan Africa	Central sub-Saharan Africa
ALB	43	Albania	Central Europe, eastern Europe and central Asia	Central Europe
AND	74	Andorra	High-income countries	Western Europe
ARE	156	United Arab Emirates	North Africa and Middle East	North Africa and Middle East
ARG	97	Argentina	High-income countries	Southern Latin America
ARM	33	Armenia	Central Europe, eastern Europe and central Asia	Central Asia
ASM	298	American Samoa	Southeast Asia, east Asia and Oceania	Oceania
ATG	105	Antigua and Barbuda	Latin America and Caribbean	Caribbean
AUS	71	Australia	High-income countries	Australasia
AUT	75	Austria	High-income countries	Western Europe
AZE	34	Azerbaijan	Central Europe, eastern Europe and central Asia	Central Asia
BDI	175	Burundi	Sub-Saharan Africa	Eastern sub-Saharan Africa
BEL	76	Belgium	High-income countries	Western Europe
BEN	200	Benin	Sub-Saharan Africa	Western sub-Saharan Africa
BFA	201	Burkina Faso	Sub-Saharan Africa	Western sub-Saharan Africa
BGD	161	Bangladesh	South Asia	South Asia
BGR	45	Bulgaria	Central Europe, eastern Europe and central Asia	Central Europe
BHR	140	Bahrain	North Africa and Middle East	North Africa and Middle East
BHS	106	Bahamas	Latin America and Caribbean	Caribbean
BIH	44	Bosnia and Herzegovina	Central Europe, eastern Europe and central Asia	Central Europe
BLR	57	Belarus	Central Europe, eastern Europe and central Asia	Eastern Europe
BLZ	108	Belize	Latin America and Caribbean	Caribbean
BMU	305	Bermuda	Latin America and Caribbean	Caribbean
BOL	121	Bolivia	Latin America and Caribbean	Andean Latin America
BRA	135	Brazil	Latin America and Caribbean	Tropical Latin America
BRB	107	Barbados	Latin America and Caribbean	Caribbean
BRN	66	Brunei	High-income countries	High-income Asia Pacific
BTN	162	Bhutan	South Asia	South Asia
BWA	193	Botswana	Sub-Saharan Africa	Southern sub-Saharan Africa
CAF	169	Central African Republic	Sub-Saharan Africa	Central sub-Saharan Africa
CAN	101	Canada	High-income countries	High-income North America
CHE	94	Switzerland	High-income countries	Western Europe
CHL	98	Chile	High-income countries	Southern Latin America
CHN	6	China	Southeast Asia, east Asia and Oceania	East Asia
CIV	205	Cote d'Ivoire	Sub-Saharan Africa	Western sub-Saharan Africa
CMR	202	Cameroon	Sub-Saharan Africa	Western sub-Saharan Africa
COD	171	Democratic Republic of the Congo	Sub-Saharan Africa	Central sub-Saharan Africa
COG	170	Congo	Sub-Saharan Africa	Central sub-Saharan Africa
COK	320	Cook Islands	Southeast Asia, east Asia and Oceania	Oceania
COL	125	Colombia	Latin America and Caribbean	Central Latin America
COM	176	Comoros	Sub-Saharan Africa	Eastern sub-Saharan Africa
CPV	203	Cabo Verde	Sub-Saharan Africa	Western sub-Saharan Africa
CRI	126	Costa Rica	Latin America and Caribbean	Central Latin America
CUB	109	Cuba	Latin America and Caribbean	Caribbean
CYP	77	Cyprus	High-income countries	Western Europe
CZE	47	Czechia	Central Europe, eastern Europe and central Asia	Central Europe
DEU	81	Germany	High-income countries	Western Europe

ISO3	location_id	location_name	Super-region	Region
DJI	177	Djibouti	Sub-Saharan Africa	Eastern sub-Saharan Africa
DMA	110	Dominica	Latin America and Caribbean	Caribbean
DNK	78	Denmark	High-income countries	Western Europe
DOM	111	Dominican Republic	Latin America and Caribbean	Caribbean
DZA	139	Algeria	North Africa and Middle East	North Africa and Middle East
ECU	122	Ecuador	Latin America and Caribbean	Andean Latin America
EGY	141	Egypt	North Africa and Middle East	North Africa and Middle East
ERI	178	Eritrea	Sub-Saharan Africa	Eastern sub-Saharan Africa
ESP	92	Spain	High-income countries	Western Europe
EST	58	Estonia	Central Europe, eastern Europe and central Asia	Eastern Europe
ETH	179	Ethiopia	Sub-Saharan Africa	Eastern sub-Saharan Africa
FIN	79	Finland	High-income countries	Western Europe
FJI	22	Fiji	Southeast Asia, east Asia and Oceania	Oceania
FRA	80	France	High-income countries	Western Europe
FSM	25	Federated States of Micronesia	Southeast Asia, east Asia and Oceania	Oceania
GAB	173	Gabon	Sub-Saharan Africa	Central sub-Saharan Africa
GBR	95	United Kingdom	High-income countries	Western Europe
GEO	35	Georgia	Central Europe, eastern Europe and central Asia	Central Asia
GHA	207	Ghana	Sub-Saharan Africa	Western sub-Saharan Africa
GIN	208	Guinea	Sub-Saharan Africa	Western sub-Saharan Africa
GMB	206	Gambia	Sub-Saharan Africa	Western sub-Saharan Africa
GNB	209	Guinea-Bissau	Sub-Saharan Africa	Western sub-Saharan Africa
GNQ	172	Equatorial Guinea	Sub-Saharan Africa	Central sub-Saharan Africa
GRC	82	Greece	High-income countries	Western Europe
GRD	112	Grenada	Latin America and Caribbean	Caribbean
GRL	349	Greenland	High-income countries	High-income North America
GTM	128	Guatemala	Latin America and Caribbean	Central Latin America
GUM	351	Guam	Southeast Asia, east Asia and Oceania	Oceania
GUY	113	Guyana	Latin America and Caribbean	Caribbean
HND	129	Honduras	Latin America and Caribbean	Central Latin America
HRV	46	Croatia	Central Europe, eastern Europe and central Asia	Central Europe
HTI	114	Haiti	Latin America and Caribbean	Caribbean
HUN	48	Hungary	Central Europe, eastern Europe and central Asia	Central Europe
IDN	11	Indonesia	Southeast Asia, east Asia and Oceania	Southeast Asia
IND	163	India	South Asia	South Asia
IRL	84	Ireland	High-income countries	Western Europe
IRN	142	Iran	North Africa and Middle East	North Africa and Middle East
IRQ	143	Iraq	North Africa and Middle East	North Africa and Middle East
ISL	83	Iceland	High-income countries	Western Europe
ISR	85	Israel	High-income countries	Western Europe
ITA	86	Italy	High-income countries	Western Europe
JAM	115	Jamaica	Latin America and Caribbean	Caribbean
JOR	144	Jordan	North Africa and Middle East	North Africa and Middle East
JPN	67	Japan	High-income countries	High-income Asia Pacific
KAZ	36	Kazakhstan	Central Europe, eastern Europe and central Asia	Central Asia
KEN	180	Kenya	Sub-Saharan Africa	Eastern sub-Saharan Africa
KGZ	37	Kyrgyzstan	Central Europe, eastern Europe and central Asia	Central Asia
KHM	10	Cambodia	Southeast Asia, east Asia and Oceania	Southeast Asia
KIR	23	Kiribati	Southeast Asia, east Asia and Oceania	Oceania
KNA	393	Saint Kitts and Nevis	Latin America and Caribbean	Caribbean
KOR	68	South Korea	High-income countries	High-income Asia Pacific
KWT	145	Kuwait	North Africa and Middle East	North Africa and Middle East
LAO	12	Laos	Southeast Asia, east Asia and Oceania	Southeast Asia
LBN	146	Lebanon	North Africa and Middle East	North Africa and Middle East

ISO3	location_id	location_name	Super-region	Region
LBR	210	Liberia	Sub-Saharan Africa	Western sub-Saharan Africa
LBY	147	Libya	North Africa and Middle East	North Africa and Middle East
LCA	116	Saint Lucia	Latin America and Caribbean	Caribbean
LKA	17	Sri Lanka	Southeast Asia, east Asia and Oceania	Southeast Asia
LSO	194	Lesotho	Sub-Saharan Africa	Southern sub-Saharan Africa
LTU	60	Lithuania	Central Europe, eastern Europe and central Asia	Eastern Europe
LUX	87	Luxembourg	High-income countries	Western Europe
LVA	59	Latvia	Central Europe, eastern Europe and central Asia	Eastern Europe
MAR	148	Morocco	North Africa and Middle East	North Africa and Middle East
MCO	367	Monaco	High-income countries	Western Europe
MDA	61	Moldova	Central Europe, eastern Europe and central Asia	Eastern Europe
MDG	181	Madagascar	Sub-Saharan Africa	Eastern sub-Saharan Africa
MDV	14	Maldives	Southeast Asia, east Asia and Oceania	Southeast Asia
MEX	130	Mexico	Latin America and Caribbean	Central Latin America
MHL	24	Marshall Islands	Southeast Asia, east Asia and Oceania	Oceania
MKD	49	North Macedonia	Central Europe, eastern Europe and central Asia	Central Europe
MLI	211	Mali	Sub-Saharan Africa	Western sub-Saharan Africa
MLT	88	Malta	High-income countries	Western Europe
MMR	15	Myanmar	Southeast Asia, east Asia and Oceania	Southeast Asia
MNE	50	Montenegro	Central Europe, eastern Europe and central Asia	Central Europe
MNG	38	Mongolia	Central Europe, eastern Europe and central Asia	Central Asia
MNP	376	Northern Mariana Islands	Southeast Asia, east Asia and Oceania	Oceania
MOZ	184	Mozambique	Sub-Saharan Africa	Eastern sub-Saharan Africa
MRT	212	Mauritania	Sub-Saharan Africa	Western sub-Saharan Africa
MUS	183	Mauritius	Southeast Asia, east Asia and Oceania	Southeast Asia
MWI	182	Malawi	Sub-Saharan Africa	Eastern sub-Saharan Africa
MYS	13	Malaysia	Southeast Asia, east Asia and Oceania	Southeast Asia
NAM	195	Namibia	Sub-Saharan Africa	Southern sub-Saharan Africa
NER	213	Niger	Sub-Saharan Africa	Western sub-Saharan Africa
NGA	214	Nigeria	Sub-Saharan Africa	Western sub-Saharan Africa
NIC	131	Nicaragua	Latin America and Caribbean	Central Latin America
NIU	374	Niue	Southeast Asia, east Asia and Oceania	Oceania
NLD	89	Netherlands	High-income countries	Western Europe
NOR	90	Norway	High-income countries	Western Europe
NPL	164	Nepal	South Asia	South Asia
NRU	369	Nauru	Southeast Asia, east Asia and Oceania	Oceania
NZL	72	New Zealand	High-income countries	Australasia
OMN	150	Oman	North Africa and Middle East	North Africa and Middle East
PAK	165	Pakistan	South Asia	South Asia
PAN	132	Panama	Latin America and Caribbean	Central Latin America
PER	123	Peru	Latin America and Caribbean	Andean Latin America
PHL	16	Philippines	Southeast Asia, east Asia and Oceania	Southeast Asia
PLW	380	Palau	Southeast Asia, east Asia and Oceania	Oceania
PNG	26	Papua New Guinea	Southeast Asia, east Asia and Oceania	Oceania
POL	51	Poland	Central Europe, eastern Europe and central Asia	Central Europe
PRI	385	Puerto Rico	Latin America and Caribbean	Caribbean
PRK	7	North Korea	Southeast Asia, east Asia and Oceania	East Asia
PRT	91	Portugal	High-income countries	Western Europe
PRY	136	Paraguay	Latin America and Caribbean	Tropical Latin America
PSE	149	Palestine	North Africa and Middle East	North Africa and Middle East
QAT	151	Qatar	North Africa and Middle East	North Africa and Middle East
ROU	52	Romania	Central Europe, eastern Europe and central Asia	Central Europe
RUS	62	Russia	Central Europe, eastern Europe and central Asia	Eastern Europe

ISO3	location_id	location_name	Super-region	Region
RWA	185	Rwanda	Sub-Saharan Africa	Eastern sub-Saharan Africa
SAU	152	Saudi Arabia	North Africa and Middle East	North Africa and Middle East
SDN	522	Sudan	North Africa and Middle East	North Africa and Middle East
SEN	216	Senegal	Sub-Saharan Africa	Western sub-Saharan Africa
SGP	69	Singapore	High-income countries	High-income Asia Pacific
SLB	28	Solomon Islands	Southeast Asia, east Asia and Oceania	Oceania
SLE	217	Sierra Leone	Sub-Saharan Africa	Western sub-Saharan Africa
SLV	127	El Salvador	Latin America and Caribbean	Central Latin America
SMR	396	San Marino	High-income countries	Western Europe
SOM	187	Somalia	Sub-Saharan Africa	Eastern sub-Saharan Africa
SRB	53	Serbia	Central Europe, eastern Europe and central Asia	Central Europe
SSD	435	South Sudan	Sub-Saharan Africa	Eastern sub-Saharan Africa
STP	215	Sao Tome and Principe	Sub-Saharan Africa	Western sub-Saharan Africa
SUR	118	Suriname	Latin America and Caribbean	Caribbean
SVK	54	Slovakia	Central Europe, eastern Europe and central Asia	Central Europe
SVN	55	Slovenia	Central Europe, eastern Europe and central Asia	Central Europe
SWE	93	Sweden	High-income countries	Western Europe
SWZ	197	Eswatini	Sub-Saharan Africa	Southern sub-Saharan Africa
SYC	186	Seychelles	Southeast Asia, east Asia and Oceania	Southeast Asia
SYR	153	Syria	North Africa and Middle East	North Africa and Middle East
TCD	204	Chad	Sub-Saharan Africa	Western sub-Saharan Africa
TGO	218	Togo	Sub-Saharan Africa	Western sub-Saharan Africa
THA	18	Thailand	Southeast Asia, east Asia and Oceania	Southeast Asia
TJK	39	Tajikistan	Central Europe, eastern Europe and central Asia	Central Asia
TKL	413	Tokelau	Southeast Asia, east Asia and Oceania	Oceania
TKM	40	Turkmenistan	Central Europe, eastern Europe and central Asia	Central Asia
TLS	19	Timor-Leste	Southeast Asia, east Asia and Oceania	Southeast Asia
TON	29	Tonga	Southeast Asia, east Asia and Oceania	Oceania
TTO	119	Trinidad and Tobago	Latin America and Caribbean	Caribbean
TUN	154	Tunisia	North Africa and Middle East	North Africa and Middle East
TUR	155	Turkey	North Africa and Middle East	North Africa and Middle East
TUV	416	Tuvalu	Southeast Asia, east Asia and Oceania	Oceania
TWN	8	Taiwan (Province of China)	Southeast Asia, east Asia and Oceania	East Asia
TZA	189	Tanzania	Sub-Saharan Africa	Eastern sub-Saharan Africa
UGA	190	Uganda	Sub-Saharan Africa	Eastern sub-Saharan Africa
UKR	63	Ukraine	Central Europe, eastern Europe and central Asia	Eastern Europe
URY	99	Uruguay	High-income countries	Southern Latin America
USA	102	United States of America	High-income countries	High-income North America
UZB	41	Uzbekistan	Central Europe, eastern Europe and central Asia	Central Asia
VCT	117	Saint Vincent and the Grenadines	Latin America and Caribbean	Caribbean
VEN	133	Venezuela	Latin America and Caribbean	Central Latin America
VIR	422	Virgin Islands	Latin America and Caribbean	Caribbean
VNM	20	Vietnam	Southeast Asia, east Asia and Oceania	Southeast Asia
VUT	30	Vanuatu	Southeast Asia, east Asia and Oceania	Oceania
WSM	27	Samoa	Southeast Asia, east Asia and Oceania	Oceania
YEM	157	Yemen	North Africa and Middle East	North Africa and Middle East
ZAF	196	South Africa	Sub-Saharan Africa	Southern sub-Saharan Africa
ZMB	191	Zambia	Sub-Saharan Africa	Eastern sub-Saharan Africa
ZWE	198	Zimbabwe	Sub-Saharan Africa	Southern sub-Saharan Africa

Appendix 2. Reconstruction of mortality and morbidity curves

All the concepts and definitions relating to life tables presented in the following have already been introduced and widely discussed in the literature. For additional details, the reader is referred to two useful handbooks by Chiang[2] and Preston et al.[3]

2.1 Mortality curves

The Global Burden of Disease (GBD) Study 2019 publishes life table data for all countries and a great diversity of regions from 1950 to 2019.[4] Specifically, it reports data on age-specific probabilities of dying and age-specific remaining life expectancy that can be used to reconstruct life tables for all region- and country-years.

GBD reports estimates in 5-year age groups, except for the first two groups of length 1 and 4, respectively. Let $A = \{0, 1, 5, 10, \dots, 105, 110\}$ be the set of starting ages of all the age groups reported by GBD. Let ${}_nq_x$ denote the unconditional probability of dying between ages x and $x + n$, where $x \in A$ and $n = 5$, except for the first two age groups in which $n = 1$ and $n = 4$, respectively. Then, the survival probability from birth to exact age x is

$$\ell_x = \prod_{y \in A, y < x} (1 - {}_nq_y) \qquad \text{(A1)}$$

with the convention that the probability of surviving from birth to age 0 is $\ell_0 = 1$ (also known as the radix of the life table). The survival curve defined in (A1) is what we refer to as 'mortality curve' and can be used to calculate life expectancy (figure 1 panel A in the main manuscript). From (A1), we can derive the distribution of ages at death, given by

$${}_nd_x = \ell_x - \ell_{x+n} \qquad \text{(A2)}$$

for all $x \in A \backslash \{110\}$. For the last age group 110+, ${}_nd_x = \ell_x$ to close the life table. Note that ${}_nd_x$ denotes the proportion of deaths between ages x and $x + n$, and that $\sum_{x \in A} {}_nd_x = 1$. The distribution of ages at death defined in (A2) can be used to measure the variability in ages at death and calculate lifespan inequality (LI) indicators (figure 1 panel C in the main manuscript).

2.2 Morbidity curves

In recent years, GBD has been publishing estimates of age- and sex-specific health-adjusted life expectancy (HALE) for all countries from 1990 to 2019.[1,5] These estimates are obtained using models that incorporate data of years lived with disability, life tables, and standard demographic methods,[1] but the underlying 'life tables in good health' remain unknown. However, if one has age-specific mortality data from a given population and makes mild assumptions of the average person-years lived in each age interval by individuals dying in that interval (the ${}_na_x$ values),[3] it is possible to reconstruct the full life table. To estimate the ${}_na_x$ values we use the following result.

Proposition. *Let* e_x *and* e_{x+n} *be the remaining life expectancies at ages* x *and* $x+n$*, respectively. Let* ${}_nq_x$ *be the unconditional probability of dying between ages* x *and* $x+n$*. Then, the average person-years lived between ages* x *and* $x+n$ *by individuals dying in that age interval is given by*

$$ {}_na_x = \frac{e_x - (e_{x+n} + n)\left(1 - {}_nq_x\right)}{{}_nq_x} \tag{A3} $$

If ${}_nq_x = 0$*, then* ${}_na_x = 0$*.*

Proof. Equation (A3) is an immediate result of the properties and relationships of the different columns of the life table. Following (A1), the probability of surviving from birth to age $x+n$ can be estimated as

$$ \ell_{x+n} = \left(1 - {}_nq_x\right)\ell_x \tag{A4} $$

provided that $\left(1 - {}_nq_x\right)$ is the probability of surviving from x to $x+n$.

Let T_x and T_{x+n} be the person-years lived above ages x and $x+n$, respectively. By definition, the remaining life expectancies at ages x and $x+n$ are

$$ e_x = \frac{T_x}{\ell_x} \qquad \text{and} \qquad e_{x+n} = \frac{T_{x+n}}{\ell_{x+n}} $$

Hence, the person-years lived between x and $x+n$ can be expressed in terms of the life expectancy and the survivorship as

$$ {}_nL_x = T_x - T_{x+n} = e_x\,\ell_x - e_{x+n}\,\ell_{x+n} \tag{A5} $$

On the other hand, let $n \cdot \ell_{x+n}$ be the person-years lived between ages x and $x+n$ by those who survived the age interval, and ${}_na_x\,(\ell_x - \ell_{x+n})$ the person-years lived by those who died. The ${}_nL_x$ values are commonly computed as the sum of these two, say

$$ {}_nL_x = n \cdot \ell_{x+n} + {}_na_x\,(\ell_x - \ell_{x+n}) = \left(n - {}_na_x\right)\ell_{x+n} - {}_na_x\,\ell_x \tag{A6} $$

Combining (A4), (A5) and (A6) yields

$$ e_x\,\ell_x - e_{x+n}\left(1 - {}_nq_x\right)\ell_x = \left(n - {}_na_x\right)\left(1 - {}_nq_x\right)\ell_x + {}_na_x\,\ell_x $$
$$ e_x - e_{x+n}\left(1 - {}_nq_x\right) = n\left(1 - {}_nq_x\right) + {}_nq_x\;{}_na_x $$
$$ {}_na_x = \frac{e_x - (e_{x+n} + n)\left(1 - {}_nq_x\right)}{{}_nq_x} $$

which proves (A3). If ${}_nq_x = 0$, then ${}_na_x = 0$ by definition, since there are no deaths between x and $x+n$. □

Corollary. *Let* e_x *and* e_{x+n} *be the remaining life expectancies at ages* x *and* $x+n$*, respectively. Let* ${}_na_x$ *be the average person-years lived between ages* x *and* $x+n$ *by individuals dying in that age interval. Then, re-arranging terms in* (A3)*, the age-specific unconditional probability of death can be estimated as*

$$ {}_nq_x = \frac{e_{x+n} + n - e_x}{e_{x+n} + n - {}_na_x} \tag{A7} $$

Equation (A7) is the key relationship that enables building a full life table using data on age-specific remaining life expectancy only, assuming data are available for all ages and that the ${}_na_x$ values are provided or can be reasonably estimated.

Using data on age-specific probabilities of dying (${}_nq_x$) and age-specific remaining life expectancy (e_x) from GBD,[4] for each country/region, sex, and year we calculated the ${}_na_x$ values by applying (A3). Next, we used the corresponding ${}_na_x$ values from each life table, in combination with the age-specific HALE estimates,[5] to reconstruct morbidity curves and the age-at-morbidity onset distributions.

Formally, let e_x^* denote the remaining health-adjusted life expectancy (HALE) at age x, and $A^* = \{0, 1, 5, 10, \dots, 90, 95\}$ the set of starting ages of all the age groups for which GBD reports HALE data.[5] In the following we add an asterisk as superscript to denote all the terms that relate to morbidity instead of mortality. Using (A7), the age-specific probabilities of health loss can be estimated as

$$ {}_nq_x^* = \frac{e_{x+n}^* + n - e_x^*}{e_{x+n}^* + n - {}_na_x} \tag{A8} $$

for all $x \in A^* \setminus \{95\}$. For the last age group 95+ we assume ${}_nq_x^* = 1$. Once these probabilities are estimated, applying analogous formulas to (A1) and (A2), we reconstructed the survival curves in good health or morbidity curves (figure 1 panel B in the main manuscript),

$$ \ell_x^* = \prod_{y \in A^*, y < x} (1 - {}_nq_y^*) \tag{A9} $$

and the age-at-morbidity onset distributions

$$ {}_nd_x^* = \ell_x^* - \ell_{x+n}^* \tag{A10} $$

As previously, $\ell_0^* = 1$ and for the last age group 95+ ${}_nd_x^* = \ell_x^*$. The ${}_nd_x^*$ values denote the proportion of individuals ceasing to be in good health between ages x and $x + n$, and can be used to measure the variability of ages-at-morbidity onset and calculate healthy lifespan inequality (HLI) indicators (figure 1 panel D in the main manuscript).

2.3 Adjustments of the ${}_na_x$ values

Equation (A8) combines the age-specific HALE estimates from GBD (e_x^*),[5] and the average person-years lived in each age interval by individuals dying in that interval (${}_na_x$) obtained by applying (A3) to GBD mortality data.[4] This equation works as long as two conditions are met for all $x \in A^* \setminus \{95\}$:

1. $e_{x+n}^* + n > e_x^*$ and $e_{x+n}^* + n > {}_na_x$, so that the probabilities are positive; and
2. $e_x^* > {}_na_x$ to ensure that the denominator is larger and ${}_nq_x^* \in (0,1)$.

Condition 1 is always met, but condition 2 is not, particularly at older ages. This is, in part, because GBD mortality estimates go up to age 110+, whereas HALE estimates end at 95+. In 5-year age groups, ${}_na_x$ values hover around 2.5 and start decreasing, approximately, at ages above 75 years. However, it may happen that at ages 75 to 95 years the remaining health-adjusted life expectancy is considerably

lower than 2.5, therefore $e_x^* < {}_na_x$ and condition 2 is not met. See, for instance, the case of Chinese males in 1990: at age 90, applying (A3) we get ${}_5a_{90} = 1.68$, but GBD reports $e_{90}^* = 1.62$. In these situations, the ${}_na_x$ values needed to be adjusted before applying (A8) to avoid ${}_nq_x^* > 1$.

From the 20,790 morbidity life tables (204 countries and territories, 19 regions, 7 super-regions, global level, 3 sex groups, and 30 years) reconstructed, in 18,038 (86.8%) of them $e_x^* > {}_na_x$ for all age groups and condition 2 was always met. Inconsistencies were detected in 2,752 (13.2%) of the cases, among which

1) In 2,050 (9.9%) of the cases the issue was solved by imputing

$$ {}_5a_{90} = \frac{{}_5a_{85} + e_{95}^*}{2} $$

   that is, in the 90−95 age group ${}_5a_{90}$ was calculated as the average between the values in the third-last and last age groups. Note that, by definition, in the last age group $e_{95}^* = {}_5a_{95}$.
2) In the remaining 702 (3.4%) life tables usually not only the second-last age group (90−95) required an adjustment. In these cases, we proceeded as follows:
   a) Using all the mortality data from GBD,[4] we calculated the average ratio ${}_na_x/e_x$ for the age groups 90−95, 95−100, 100−105, and 105−110 (from the fifth-last to the second-last):

	**90−95**	**95−100**	**100−105**	**105−110**
${}_na_x/e_x$	0.563	0.673	0.771	0.846

   b) Next, we applied these ratios to calculate ${}_na_x$ from e_x^* whenever inconsistencies were detected and ensure that $e_x^* > {}_na_x$. The estimated ratio of the 105−110 age group was applied to the second-last group (90−95) of the morbidity life tables, and so on.

Appendix 3. Inequality measures and uncertainty

3.1 The standard deviation as a measure of inequality

Using life table notation, the standard deviation of an age-at-death distribution beginning at age x is defined as

$$SD_x = \sqrt{\frac{1}{\ell_x} \sum_{y \in A, y \geq x} {}_nd_y \left(y + {}_na_y - x - e_x\right)^2} \quad \text{(A11)}$$

where x is the age at which the age-at-death distribution starts (in this paper we only report for $x = 0$ and $x = 65$), ℓ_x is the initial life table population at age x, e_x is the remaining life expectancy at age x, and ${}_nd_y$ and ${}_na_y$ are, respectively, the proportion of deaths and the average-person years lived in the interval by those dying in the interval (or average age at death in the interval) between y and $y + n$. This is a very popular and basic indicator that measures the variability in the ages at death around the mean of the distribution, and that we adopt as a lifespan inequality (LI) indicator.

The same formula can be applied to an age-at-morbidity onset distribution (whose derivation is explained in Appendix 2) to calculate the corresponding level of healthy lifespan inequality (HLI). Thus, the standard deviation of the age-at-morbidity onset distribution beginning at age x is calculated as

$$SD_x^* = \sqrt{\frac{1}{\ell_x^*} \sum_{y \in A^*, y \geq x} {}_nd_y^* \left(y + {}_na_y - x - e_x^*\right)^2} \quad \text{(A12)}$$

where ℓ_x^* is the initial life table population in good health, e_x^* is the health-adjusted remaining life expectancy (HALE) at age x, and ${}_nd_y^*$ is the proportion of individuals ceasing to be in good health between ages y and $y + n$. The HLI indicator SD_x^* measures the variability in individuals' healthy lifespans.

3.2 Other inequality measures

We compared our lifespan inequality (LI) and healthy lifespan inequality (HLI) measures based on the standard deviation with those derived from using the coefficient of variation (CoV) and the Gini coefficient (G) as inequality measures by applying the following formulas:

$$CoV_x = \frac{SD_x}{e_x + x} \qquad CoV_x^* = \frac{SD_x^*}{e_x^* + x}$$

$$G_x = \frac{1}{2(\ell_x)^2} \sum_{y \in A, y \geq x} \sum_{z \in A, z \geq x} {}_nd_y \, {}_nd_z \left|\left(y + {}_na_y\right) - \left(z + {}_na_z\right)\right|$$

$$G_x^* = \frac{1}{2(\ell_x^*)^2} \sum_{y \in A^*, y \geq x} \sum_{z \in A^*, z \geq x} {}_n d_y^* \, {}_n d_z^* \left| \left(y + {}_n a_y\right) - \left(z + {}_n a_z\right) \right|$$

The coefficient of variation is simply the relative version of the standard deviation, while the Gini coefficient is a very popular index of inequality that measures the expected difference between two randomly chosen observations.

As shown in Figs. S1 and S2, all measures of HLI are highly correlated ($r > 0.91$), both for females and males.

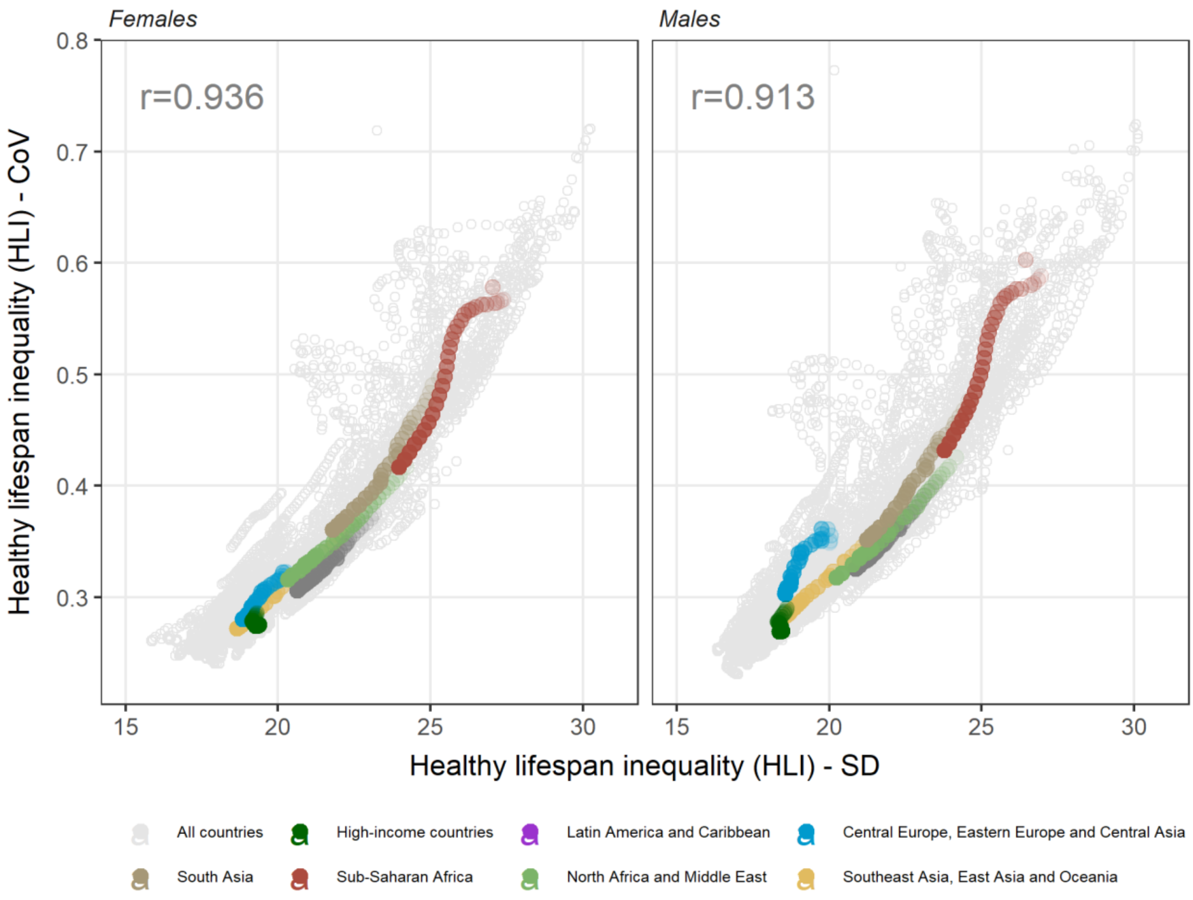

Figure S1. Relationship between healthy lifespan inequality measured with the standard deviation and healthy lifespan inequality measured with the coefficient of variation by sex between 1990 and 2019. For each region, the lightest colour corresponds to 1990 and darkness increases over time up to 2019; r denotes the correlation coefficient between these measures across all countries. The outlier dots representing Rwanda 1994 are not shown. Source: Authors' elaboration based on GBD data.[4,5]

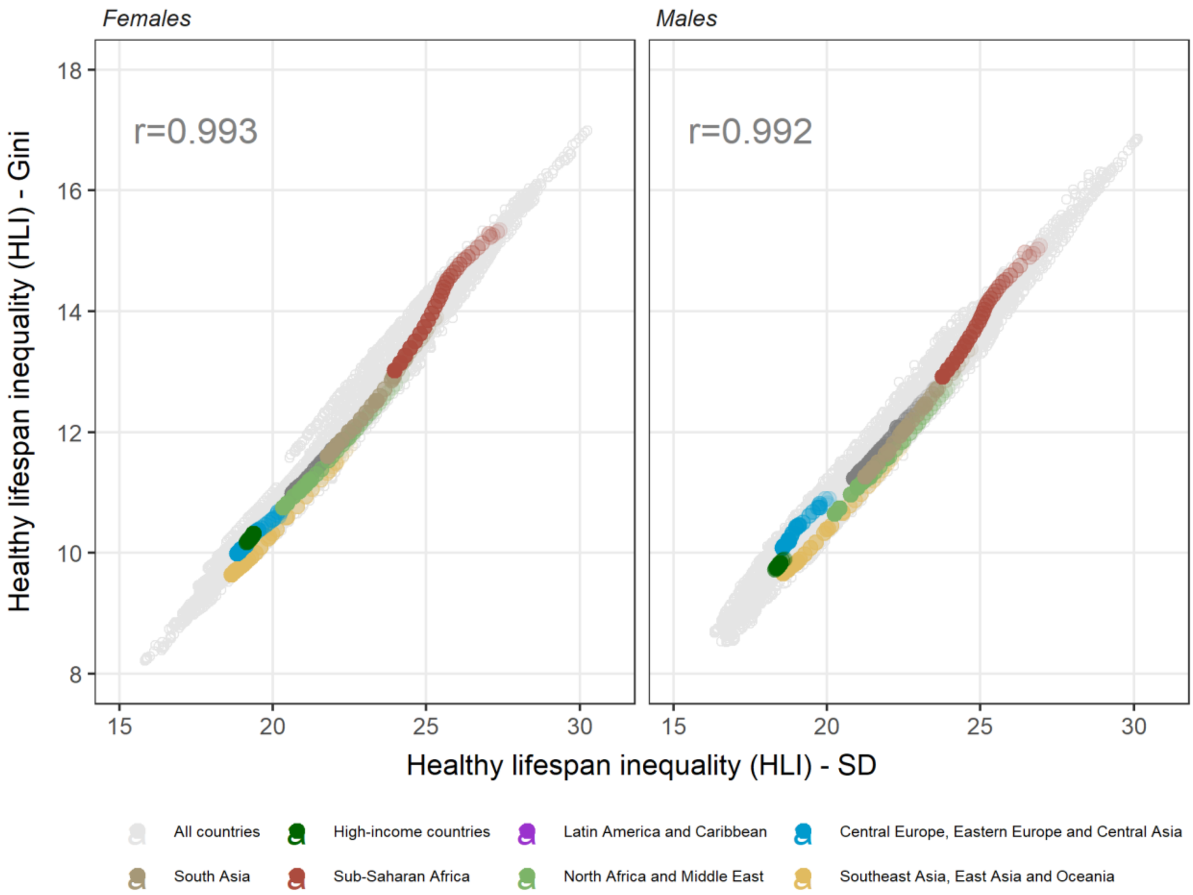

Figure S2. Relationship between healthy lifespan inequality measured with the standard deviation and healthy lifespan inequality measured with the Gini index by sex between 1990 and 2019. For each region, the lightest colour corresponds to 1990 and darkness increases over years up to 2019; r denotes the correlation between these measures across countries. The outlier dots representing Rwanda 1994 are not shown. Source: Authors' elaboration based on GBD data.[4,5]

3.3 Uncertainty estimation

We assessed the uncertainty of the LI and HLI estimates based on the uncertainty of the input data from GBD. Uncertainty was obtained by sampling from the corresponding 95% uncertainty intervals of life expectancy (e_x), death probabilities (${}_nq_x$), and HALE (e_x^*) reported by GBD on Monte Carlo simulations, applying a similar approach than used elsewhere.[6]

We assumed GBD data are normally distributed with mean values equal to the point estimates. For each country/region, sex, and year we proceeded as follows:

1) For each age-specific estimate (e_x, ${}_nq_x$, and e_x^*), we approximated the standard deviation by dividing the range of the corresponding 95% uncertainty interval by 3.92.
2) We randomly drew 10,000 samples of age-specific e_x, ${}_nq_x$, and e_x^* from a truncated normal distribution with means equal to the point estimates and corresponding standard deviations:
   a) For e_x and e_x^* we set a lower bound of 0 to only have positive values, with no upper limit.
   b) For ${}_nq_x$ the distribution was truncated between 0 and 1.

3) For each draw of age-specific e_x and ${}_nq_x$, we applied (A1) an (A2) to calculate 10,000 sets of survival curves (ℓ_x) and age-at-death distributions (${}_nd_x$).
4) From the sets of ${}_nd_x$, we applied (A11) to obtain 10,000 estimates of SD_x at ages $x = 0$ and $x = 65$, from which we calculated the corresponding 80% uncertainty intervals of the LI levels reported in the paper.
5) Besides, for each draw of age-specific e_x^*, we applied (A8) to calculate 10,000 sets of age-specific probabilities of health loss (${}_nq_x^*$).
   a) We did not incorporate any uncertainty on the ${}_na_x$ values, as this generated too much noise. We used the point estimates of ${}_na_x$ calculated as described in Appendices 2.2 and 2.3.
   b) When applying (A8) to random draws of e_x^* we faced a new challenge: Condition 1 in Appendix 2.3 was not always met. Due to randomness, it may happen that $e_{x+n}^* + n < e_x^*$ or $e_{x+n}^* + n < {}_na_x$. As a result, ${}_nq_x^*$ could take any values and were not only restricted to the interval (0,1).
   c) To address this issue, we re-sampled ${}_nq_x^*$ from a log-normal distribution.
      i) For each sample of age-specific ${}_nq_x^*$ obtained by applying (A8) we calculated its mean m and its standard deviation s.
      ii) We then used standard formulae to obtain the usual log-normal parameters mean μ and variance σ^2, given by

$$\mu = \log\left(\frac{m^2}{\sqrt{s^2 + m^2}}\right) \quad \text{and} \quad \sigma^2 = \log\left(1 + \frac{s^2}{m^2}\right)$$

      iii) By drawing random values from a truncated log-normal distribution with upper bound set at 1 and these μ and σ^2 parameters we ensured ${}_nq_x^* \in (0,1)$.
6) Next, we applied (A9) and (A10) to calculate 10,000 sets of morbidity curves (ℓ_x^*) and age-at-morbidity onset distributions (${}_nd_x^*$).
7) From the sets of ${}_nd_x^*$, we applied (A12) to obtain 10,000 estimates of SD_x^* at ages $x = 0$ and $x = 65$, from which we calculated the corresponding 80% uncertainty intervals of the HLI levels reported in the paper.

3.4 Transparency and replicability

We carried out our analyses using the open-source statistical software R (version 4.1.1).[7] The source code to replicate the analyses, input data, and results are publicly available for research purposes on the GitHub repository https://github.com/panchoVG/HLI.

Appendix 4. The GATHER checklist

Item #	Checklist item	Reported on page #
**Objectives and funding**		
**1**	Define the indicator(s), populations (including age, sex, and geographic entities), and time period(s) for which estimates were made.	Methods; Appendix 1
**2**	List the funding sources for the work.	Acknowledgements
**Data Inputs**		
For all data inputs from multiple sources that are synthesized as part of the study:		
**3**	Describe how the data were identified and how the data were accessed.	Methods
**4**	Specify the inclusion and exclusion criteria. Identify all ad-hoc exclusions.	Not Applicable
**5**	Provide information on all included data sources and their main characteristics. For each data source used, report reference information or contact name/institution, population represented, data collection method, year(s) of data collection, sex and age range, diagnostic criteria or measurement method, and sample size, as relevant.	Methods; Appendix 1; Appendix 2
**6**	Identify and describe any categories of input data that have potentially important biases (e.g., based on characteristics listed in item 5).	Discussion
For data inputs that contribute to the analysis but were not synthesized as part of the study:		
**7**	Describe and give sources for any other data inputs.	Not Applicable
For all data inputs:		
**8**	Provide all data inputs in a file format from which data can be efficiently extracted (e.g., a spreadsheet rather than a PDF), including all relevant meta-data listed in item 5. For any data inputs that cannot be shared because of ethical or legal reasons, such as third-party ownership, provide a contact name or the name of the institution that retains the right to the data.	Appendix 3.4 (GitHub repository)
**Data analysis**		
**9**	Provide a conceptual overview of the data analysis method. A diagram may be helpful.	Methods
**10**	Provide a detailed description of all steps of the analysis, including mathematical formulae. This description should cover, as relevant, data cleaning, data pre-processing, data adjustments and weighting of data sources, and mathematical or statistical model(s).	Appendix 2; Appendix 3
**11**	Describe how candidate models were evaluated and how the final model(s) were selected.	Appendix 3.2
**12**	Provide the results of an evaluation of model performance, if done, as well as the results of any relevant sensitivity analysis.	Appendix 3.2
**13**	Describe methods for calculating uncertainty of the estimates. State which sources of uncertainty were, and were not, accounted for in the uncertainty analysis.	Appendix 3.3
**14**	State how analytic or statistical source code used to generate estimates can be accessed.	Data sharing; Appendix 3.4
**Results and Discussion**		
**15**	Provide published estimates in a file format from which data can be efficiently extracted.	Appendices 5 and 6 (GitHub repository)
**16**	Report a quantitative measure of the uncertainty of the estimates (e.g. uncertainty intervals).	Results; Appendices 5 and 6
**17**	Interpret results in light of existing evidence. If updating a previous set of estimates, describe the reasons for changes in estimates.	Results: Discussion
**18**	Discuss limitations of the estimates. Include a discussion of any modelling assumptions or data limitations that affect interpretation of the estimates.	Discussion

Appendix 5. Lifespan inequality and healthy lifespan inequality estimates

Global, regional, and national estimates of lifespan inequality (LI) and healthy lifespan inequality (HLI) for the period 1990−2019, and the corresponding 80% uncertainty intervals, are available on the GitHub repository https://github.com/panchoVG/HLI.

The file ‘EstimatesHLI-LI-1990-2019.csv’ containing all the estimates has the following variables/columns:

- location_id: Location number assigned by GBD
- location_name: Name of the location
- ISO3: Three-letter country codes (only for countries/territories)
- Type: Whether the location is a super-region, super-region/region, region, or country/territory
- SuperRegion: Super-region at which a region or country/territory belong
- Region: Region at which a country/territory belongs
- Year: 1990−2019
- Sex: Female, male, or both sexes combined
- Age: 0 for estimates that refer to all ages, and 65 for estimates that refer to ages above 65 years
- Measure: ‘Mortality’ for estimates of lifespan inequality, and ‘Morbidity’ for estimates of healthy lifespan inequality
- ex: Corresponding values of life expectancy or HALE reported by GBD
- SD: Estimated level of lifespan inequality/healthy lifespan inequality, measured as standard deviation of the corresponding age-at-death/age-at-morbidity onset distribution
- SDlow10: Lower bound (10% percentile) of the estimated standard deviation of the corresponding age-at-death/age-at-morbidity onset distribution
- SDup90: Upper bound (90% percentile) of the estimated standard deviation of the corresponding age-at-death/age-at-morbidity onset distribution

Appendix 6. Global and regional HLI/LI ratios

Global, regional, and national estimates of the ratio between healthy lifespan inequality and lifespan inequality (HLI/LI) for the period 1990−2019, and the corresponding 80% uncertainty intervals, are available on the GitHub repository https://github.com/panchoVG/HLI
(file 'RatiosHLI-LI-1990-2019.csv').

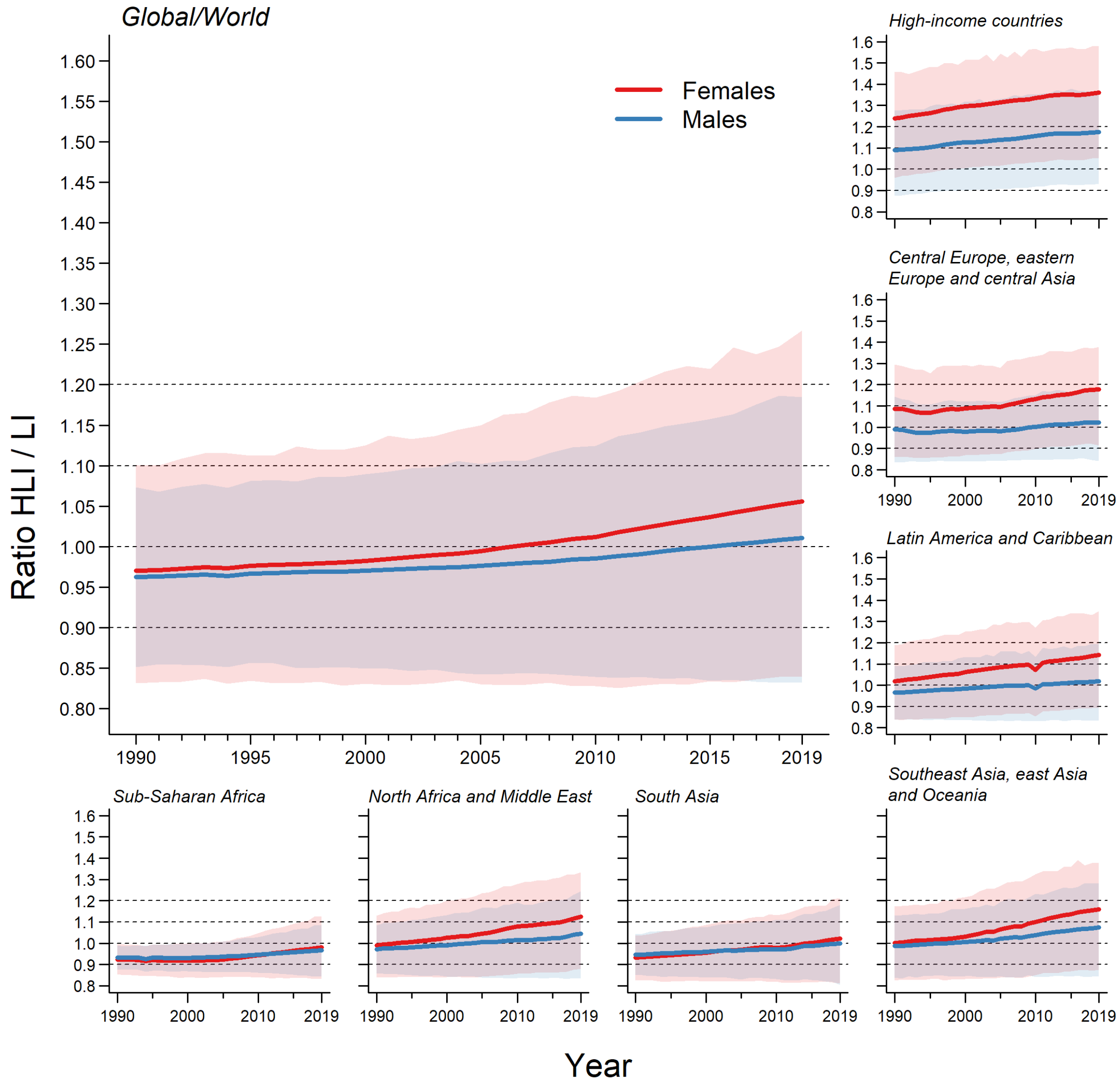

Figure S3. Global and regional trends in the ratio between healthy lifespan inequality (HLI) and lifespan inequality (LI) by sex, 1990−2019. Shadowed areas represent 80% uncertainty intervals. Source: Authors' elaboration based on GBD data.[4,5]

References

1. Wang H, Abbas KM, Abbasifard M, *et al.* Global age-sex-specific fertility, mortality, healthy life expectancy (HALE), and population estimates in 204 countries and territories, 1950–2019: a comprehensive demographic analysis for the Global Burden of Disease Study 2019. *The Lancet* 2020; **396**: 1160–203.
2. Chiang, C. L. *The life table and its applications*. Malabar, FL: Robert E. Krieger, 1984.
3. Preston, S. H., P. Heuveline, and M. Guillot. *Demography: Measuring and modeling population processes*. Oxford: Blackwell, 2001.
4. Global Burden of Disease Collaborative Network. Global Burden of Disease Study 2019 (GBD 2019) Life Tables 1950-2019. Seattle, United States of America: Institute for Health Metrics and Evaluation (IHME), 2020. Available from: http://ghdx.healthdata.org/record/ihme-data/gbd-2019-life-tables-1950-2019 (accessed July 11, 2022).
5. Institute for Health Metrics and Evaluation. GBD Results Tool. Global Health Data Exchange. Available from: http://ghdx.healthdata.org/gbd-results-tool (accessed July 11, 2022).
6. Villavicencio F, Bergeron-Boucher M-P, Vaupel JW. Reply to Permanyer et al.: The uncertainty surrounding healthy life expectancy indicators. *Proc Natl Acad Sci* 2021; **118**: e2115544118.
7. R Core Team. R: A language and environment for statistical computing. R Foundation for Statistical Computing. 2021. https://www.R-project.org/.
